# Modeling analysis reveals the transmission trend of COVID-19 and control efficiency of human intervention

**DOI:** 10.1186/s12879-021-06560-3

**Published:** 2021-08-21

**Authors:** Chaoyuan Cheng, Xinru Wan, Zhibin Zhang

**Affiliations:** 1 State Key Laboratory of Integrated Management on Pest Insects and Rodents in Agriculture, Institute of Zoology, Chinese Academy of Sciences, Beijing, 100101 China; 2 CAS Center for Excellence in Biotic Interactions, University of Chinese Academy of Sciences, Beijing, 100049 China; 3 University of Chinese Academy of Sciences, Beijing, 100049 China

**Keywords:** COVID-19, SARS-CoV-2, Spread pattern, Control efficiency

## Abstract

**Background:**

A novel coronavirus disease (COVID-19) has caused huge damage to public health around the world. Revealing the transmission dynamics of COVID-19 and control efficiency is important for containing the spread of the virus.

**Methods:**

By using a logistic growth model, we estimated the transmission parameters of COVID-19 in China and six other countries (Republic of Korea, Iran, Italy, Spain, France and Germany). The transmission parameters represent the maximum daily increase rate in the early stages of the epidemic and the control efficiency under human intervention. The control efficiency was determined by the significant decrease of the daily increase rate in time and cumulative cases.

**Results:**

We found the daily increase rate of cumulative cases of COVID-19 decreased significantly in both time and cumulative cases in all countries, but the decreasing trend was not further reduced in other countries except for China and Republic of Korea. The response of the daily increase rate to control measures was much earlier than the number of new cases.

**Conclusions:**

Our results suggested that lockdown at the epicenter and social distancing effectively reduced the spread of COVID-19 in the early stage, but identification and isolation of patients, suspected cases and people with close contact at a community level is essential in further reduction of the daily increase rate of COVID-19.

**Supplementary Information:**

The online version contains supplementary material available at 10.1186/s12879-021-06560-3.

## Background

Recently, a novel coronavirus (defined as SARS-CoV-2 by the International Committee on Taxonomy of Viruses) emerged as a serious threat to the public health in China and around the world. Patients infected by the virus showed typical clinical symptoms of fever, dry cough, dyspnea, headache, and pneumonia (a disease defined as COVID-19 by World Health Organization), which could result in respiratory failure or even death [1]. A total of 1,521,252 confirmed cases were reported globally, with 92,798 deaths by April 10, 2020. The first unknown pneumonia cases in China were noted in a local food market in Wuhan, the capital of Hubei Province, on December 29, 2019 [2]. The discovery was reported to WHO and its national members on January 3, 2020. The genome of the virus was sequenced and identified as a novel coronavirus by Chinese scientists on January 7 [2, 3].

On January 20, China’s National Health Commission listed the COVID-19 as the No. 1 infectious disease for prevention and control in China, and after this date, both central and local governments started first-class actions for a public health emergency. An unprecedented lockdown of Wuhan, a city with over 10 million people, was implemented on January 23. A series of control measures, such as travel restrictions and lockdown of the epicenter, isolation and observation of suspected or infected patients or places at various scales were taken in China. However, there were concerns about the effectiveness or necessity of these measures taken for controlling the COVID-19 in China, and whether or not the virus could be well contained [4]. These concerns were soon removed with the successful control of the spread of COVID-19 in China. With the fast expansion of COVID-9 around the world, most countries affected have adopted similar quarantine measures as those taken by China to contain the spread of COVID-19.

We searched PubMed [5] and medRxiv [6] for studies about COVID-19 published in English up to March 31, 2020, and identified 177 and 315 related results, respectively. There were 50 studies (13 PubMed, 37 medRxiv) on control methods and efficiency of COVID-19, 21 studies (7 PubMed, 14 medRxiv) on the influence of travel or migration on the spread of SARS-CoV-2 and 69 (11 PubMed, 58 medRxiv) studies on the estimation of epidemiological parameter (i.e. incubation and basic reproductive rate) or growth of cumulative cases. There were two studies using the logistic growth model. In these previous studies, the control efficiency was mainly evaluated by Susceptible-Exposed-Infectious-Recovered (SEIR) models under different control scenarios. No study was found to use the relationship between daily increase rate and time or cumulative cases to assess the transmission trend and control efficiency of COVID-19.

Revealing the transmission trend of COVID-19 and assessing the efficiency of the control measures employed by different countries is important to reduce the spread of COVID-19. Although the epidemiological properties of COVID-19 have been investigated by using modelling approaches [7–12], knowledge about its influencing factors and efficiency of current control measures is still limited (but see [8, 12]). Recently, several papers have been published to assess the efficiency of these control measures using SIER models under various scenarios [12–14]. However, differences of control efficiency on COVID-19 between different countries have not been fully investigated.

Here, based on official data released on April 10 concerning patients infected with the COVID-19 virus in China and the WHO data of some selected countries that were heavily hit by COVID-19 (the cumulative cases was larger than 10,000 by April 3) that showed an obvious decrease of new cases [15], we assessed the transmission trend of COVID-19 and control efficiency of several countries (including China, R. Korea, Iran, Italy, Spain, France and Germany) by using a logistic growth model. By referring to our previous study on SARS [16], the cumulative number of COVID-19 cases could be described by a logistic model under human intervention. It was expected that the daily increase rate of cumulative cases of COVID-19 should decrease against time and cumulative cases under the effective control of human intervention.

We found the relationship between the daily increase rate and cumulative cases or time can be used to assess the transmission trend and control efficiency of COVID-19. All countries showed a similar decreasing trend of daily increase rate in the early stage, but only China and R. Korea showed a sustained decline of daily increase rate in the later stage. The difference might be caused by the different control strategies at individual and community levels employed by different countries.

## Methods

### Data sources

**Fig. 1:**
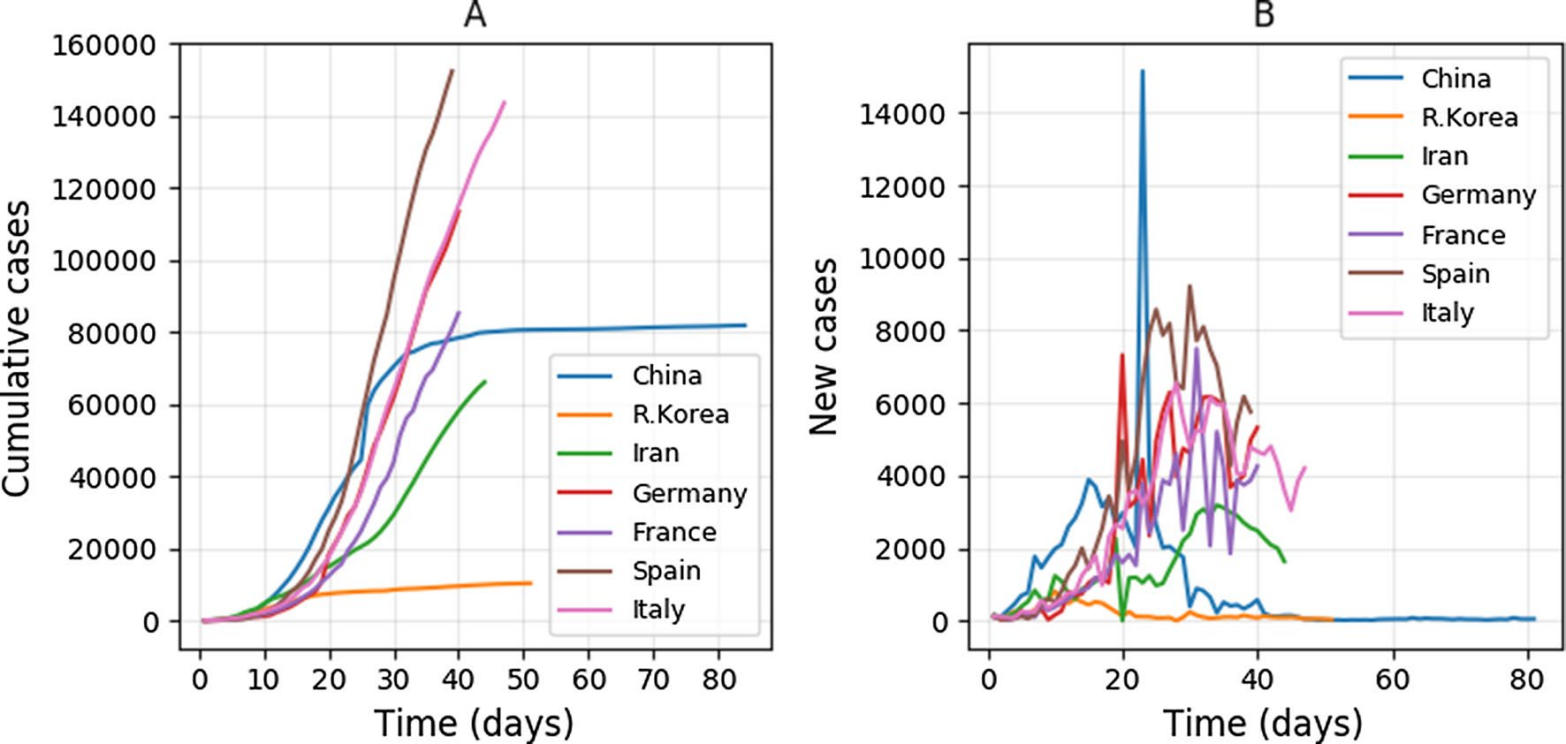
The temporal change of cumulative cases (**A**) and new cases (**B**) of COVID-19 in the seven countries. For sake of comparison, the starting day of the epidemic in all countries was set to zero when the cumulative number of cases was larger and close to 100

We complied original data of the cumulative cases of infected patients from January 10, 2020 to April 10, 2020 based on the official website of the central and local provincial governments in the mainland of China (Fig. 1, Additional file 1: Data S1), and epidemiological data of some cases that travelled to Wuhan from open access data [17]. The data covered the main period of the epidemic in China [18]. We obtained data of the cumulative cases of COVID-19 of R. Korea, Iran, Italy, Spain, France and Germany from the WHO website [15].

### Incubation time and infection time estimation

In order to calculate basic reproduction numbers of COVID-19 from the daily increase rate (see below), we needed to calculate the incubation and infection time of COVID-19. Using published epidemiological case data [19], we edited a subset of data concerning exported cases from Wuhan to other recipient cities in China (Additional file 2: Table S1). We used only data with detailed information including age, gender, date of onset, and travel information. We estimated the incubation time (IBT) by calculating the time difference between the arrival date of the patient from Wuhan to the date when the patient showed symptoms of COVID-19, and the infection time (IFT) by calculating the time difference between the arrival date from Wuhan to the date when the patient was hospitalized outside of Wuhan. The arrival date to Wuhan was assumed to be the start time of exposure by patients to COVID-19. We excluded the epidemiological data if IBT ≤ 0 or ≥ 20 in calculating IBT and IFT due to uncertainty of the data or influence on these estimates of the majority of patients. We did not use the data of Wuhan residents for calculating IBT or IFT because we had no information when they first contacted a patient or the virus.

### Model structure of linear logistic growth model

The exponential model is suitable for fitting the population growth of infected patients without human intervention. It is true in the very early stage of disease transmission. With increase of the number of infected patients, prevention and control measures would be taken by people and the government which would reduce the transmission ability. Thus, a logistic model was suitable for fitting the population growth of infected patients under human intervention. By referring to Zhang et al. (2004) [16], the model describing the population growth of cumulative cases of COVID-19 was defined as:

1 $$N_{t + 1} = N_{t} e^{{r_{m} \left( {1 - \frac{{N_{t} }}{K}} \right)}}$$

${N}_{t}$ is the number of cumulative cases at day *t*, *K* is the maximum population size infected by a disease under human intervention. *r*_*m*_ is the maximum daily increase rate. From Eq. 1, the daily increase rate (*r*_*t*_) of the cumulative cases of patients was defined as follows:

2 $$r_{t} = \ln \left( {\frac{{N_{t + 1} }}{{N_{t} }}} \right) = r_{m} \left( {1 - \frac{{N_{t} }}{K}} \right)$$

Thus, it is expected that, under human intervention, the daily increase rate should be negatively associated with the number of cumulative cases of patients [16]. The relationship between daily increase rate and the number of cumulative cases was rewritten as follows:

3 $$r_{t} = a + bN_{t}$$

Here, *a, b* are constants, and all > 0 (Additional file 2: Fig. S1). *a* represents the maximum daily increase rate (*r*_*m*_), $b= -\frac{{r}_{m}}{K}=-\frac{a}{K}$. Parameter *a* represents the maximum daily increase rate when the cumulative cases are close to zero without or with little human intervention because very few cases often cause little attention of disease prevention or control to people and governments. Parameter *b* represents the control efficiency which measures the reduction speed of transmission ability under human intervention. According to Zhang (2004), the inflection point of the logistic model is the number of infections when it reaches to K/2. Thus, estimation of K could help to predict the inflection point of disease transmission.

### Model structure of nonlinear logistic growth model

If the relationship between the daily increase rate and cumulative cases is nonlinear, Eq. 3 can be modified into the following formula:

4 $$r_{t} = a + bN_{t}^{c}$$

Here, *c* is a constant. If *c* = 1, the relation is linear, otherwise, it is a nonlinear. Non-significant association between *r*_*t*_ and *N*_*t*_ means the disease spread without the effective control.

Similarly, it is also expected that the daily increase rate would decrease in time under the effective human intervention which can be described by referring to Eqs. 3 and 4.

## Results

Of the seven countries we studied, the number of cumulative cases and new cases showed almost zero-growth in the mainland of China and Republic of Korea, while the other countries only showed signs of a steady decrease of new cases (Fig. 1). The order of control efficiency as measured by the date when the number of new COVID-19 cases showed obvious decrease was: R. Korea (day 9) > China (day 15, excluding the value on February 12 due to including the clinic cases) > Germany (day 26) > Italy (day 27) > Spain (day 30) > France (day 34) > Iran (day 38) (Fig. 1B), indicating the control efficiency against time was high for R. Korea and China.

The daily increase rate of cumulative cases of all countries was significantly and negatively associated with the cumulative cases and time (all *p* < 0.001, Table 1), indicating the control measures in these countries were all effective. The rank of regression coefficients against cumulative case was: R. Korea > Iran > China > France > Germany > Spain > Italy; the ranks of the regression coefficient against time was: R. Korea > China > Spain > Iran > France > Italy > Germany (Table 1), indicating the control efficiency against cumulative cases was high for R. Korea and Iran, and the control efficiency against time was high for R. Korea and China.

**Table 1:** Fitting results on the relation between the daily increase rate and the number of cumulative cases using the linear Eq. 3

Countries	Cumulative cases				Time (days)			
	*a*	*b* (× 10^5^)	*p*	r^2^	*a*	*b*	*p*	r^2^
China	0.3239	− 0.4281	0.0000	0.79	0.3656	− 0.0098	0.0000	0.77
R. Korea	0.3886	− 4.4526	0.0000	0.79	0.3208	− 0.0103	0.0000	0.60
Italy	0.2482	− 0.1964	0.0000	0.57	0.3329	− 0.0080	0.0000	0.73
Spain	0.2807	− 0.2004	0.0000	0.64	0.3624	− 0.0093	0.0000	0.69
Germany	0.2506	− 0.2332	0.0000	0.37	0.3149	− 0.0074	0.0000	0.37
France	0.2494	− 0.3134	0.0000	0.44	0.3338	− 0.0085	0.0000	0.59
Iran	0.2465	− 0.4533	0.0000	0.40	0.3303	− 0.0089	0.0000	0.60

*a* and *b* are the model coefficients defined in Eq. 3. *p* represents the significance of the model fit and r^2^ represents goodness of the fit

**Fig. 2:**
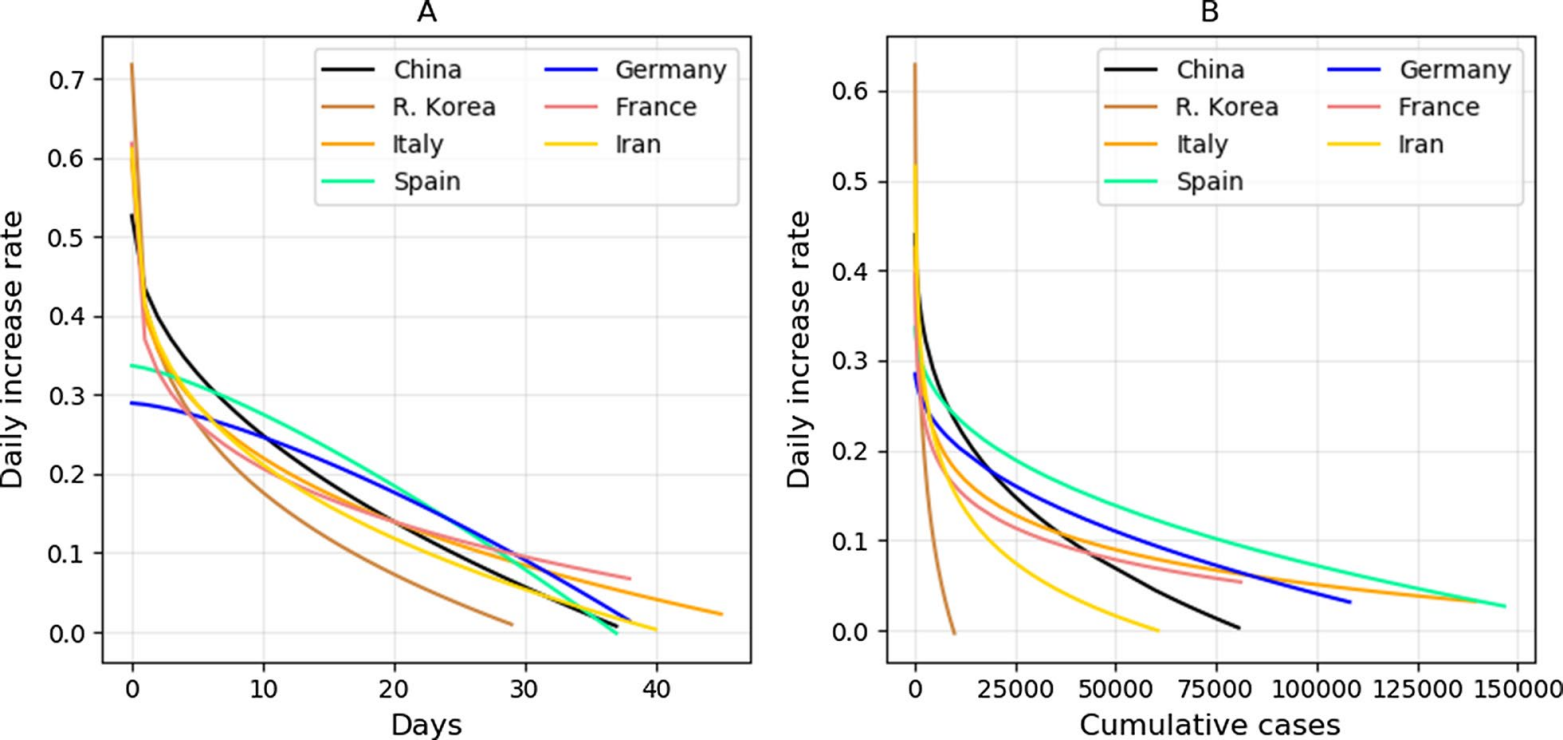
Fitting results on relation between the daily increase rate of cumulative cases and time (**A**) or cumulative cases (**B**) using nonlinear Eq. 4 (also see Table 2). In theory, the daily increase rate should be greater or equal to zero. Thus, we did not show the negative values produced by model fitting

There was strong nonlinearity between the daily increase rate and cumulative cases in some countries. By referring to the increased percentage of variance explained using nonlinear model (Eq. 4) than using linear model (Eq. 3), the daily increase rate with cumulative cases showed strong convex relation with cumulative cases for Italy (+ 44%), France (+ 46%) and Iran (+ 116%), and with time for R. Korea (+ 45%) and Iran (+ 34%) (Table 2, Fig. 2), indicating the control efficiency in these countries was reduced in the late stage as compared to that in the early stage. Figure 2 clearly demonstrated that R. Korea, China and Iran performed better in reducing the transmission of COVID-19 as measured by decrease of daily increase rate against time and/or the number of cumulative cases.

**Table 2:** Fitting results on the relation between daily increase rate and cumulative cases using the nonlinear Eq. 4

Countries	Cumulative cases				Time (days)			
	*a*	*b*	*c*	r^2^	*a*	*b*	*c*	r^2^
China	0.52	− 0.0223	0.2792	0.88 (+ 13%)	0.5263	− 0.0921	0.4786	0.83 (+ 9%)
R. Korea	912.2	− 911.0	0.0002	0.94 (+ 20%)	0.7173	− 0.3019	0.2529	0.87 (+ 45%)
Italy	809.1	− 808.4	0.0001	0.82 (+ 44%)	0.5964	− 0.1969	0.2809	0.87 (+ 18%)
Spain	0.36	− 0.0035	0.3818	0.72 (+ 13%)	0.3368	− 0.0030	1.3051	0.70 (+ 1%)
Germany	0.30	− 0.0014	0.4537	0.40 (+ 7%)	0.2895	− 0.0017	1.3921	0.38 (+ 2%)
France	308.4	− 307.8	0.0002	0.64 (+ 46%)	0.6179	− 0.2482	0.2189	0.70 (+ 20%)
Iran	1657.5	− 1656.5	0.0001	0.86 (+ 116%)	0.6108	− 0.1982	0.3035	0.81 (+ 34%)

*a*, *b* and *c* are the model coefficients defined in Eq. 4. r^2^ represents goodness of the fit. The percentage after r^2^ was the increased percentage of variance explained by the nonlinear model than those using linear model (Table 1)

**Fig. 3:**
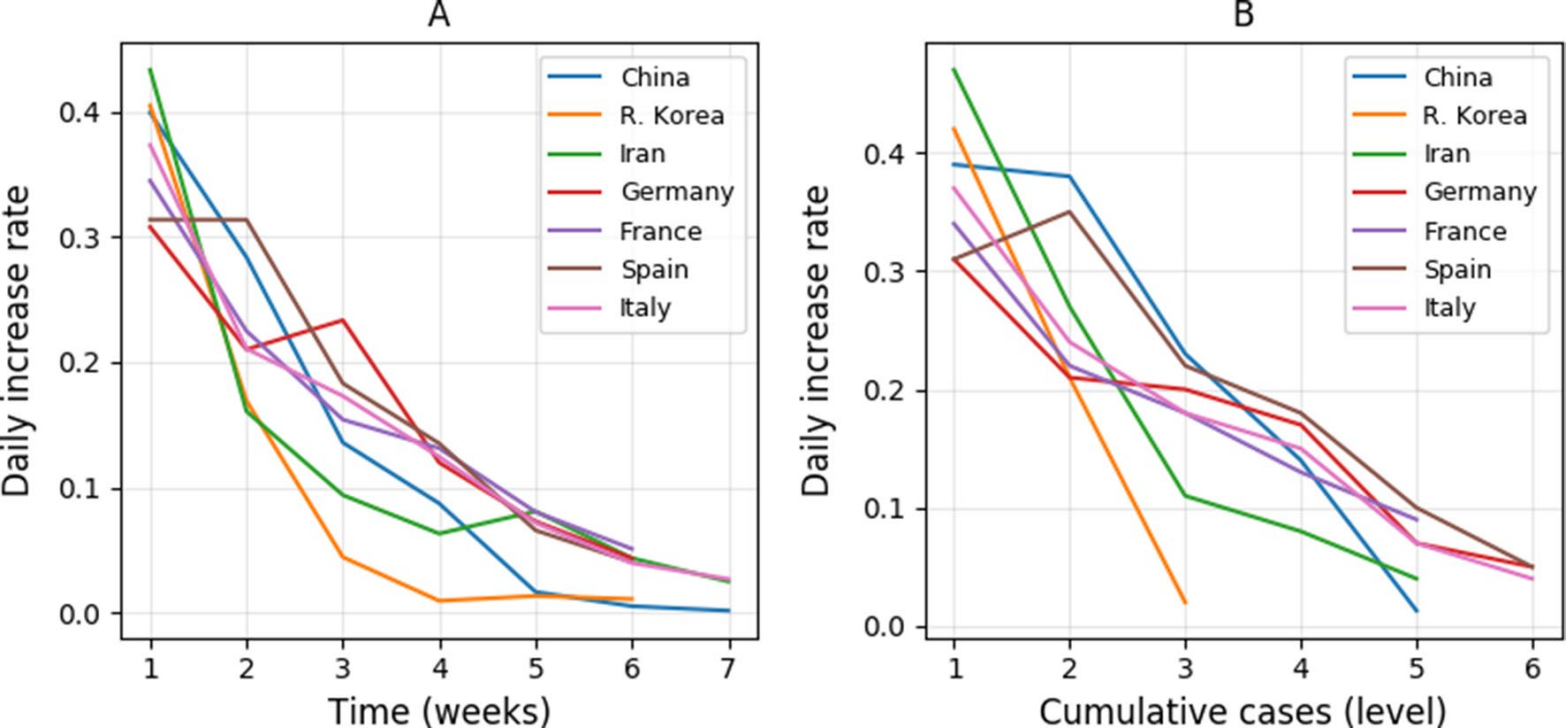
Comparisons of the changing trend of the average daily increase of cumulative cases of COVID-19 of seven countries against time (**A**) and against cumulative cases (**B**). A, the average daily increase rate for every week. B, the average daily increase rate for different levels of cumulative cases: 1. 100–1000, 2. 1000–5000, 3. 5000–10,000, 4. 10000–50,000, 5. 50,000–100,000, 6. > 100,000. The initial daily increase rate of all countries was calculated starting from the cumulative cases larger than and close to 100

Figure 3 showed the different changing trend of daily increase rate against time and cumulative cases among these seven countries at different levels of weeks or cumulative cases. All countries had a similar daily increase rate (around 0.3–0.4) in the 1st week. All countries showed a steady decrease of daily increase rate in time and against cumulative cases. However, China and R. Korea reduced the daily increase rate close to zero by week 5 or 6. All countries had a similar decreasing trend of daily increase rate during the first 2 weeks, but the daily increase rate was not reduced as much as in the following weeks in the other countries except for China and R. Korea. From Fig. 3, the changing trend of daily increase rate against time and cumulative cases could help us roughly estimate the end date of an epidemic and the maximum number of cumulative cases, if control efforts and efficiency were maintained.

**Fig. 4:**
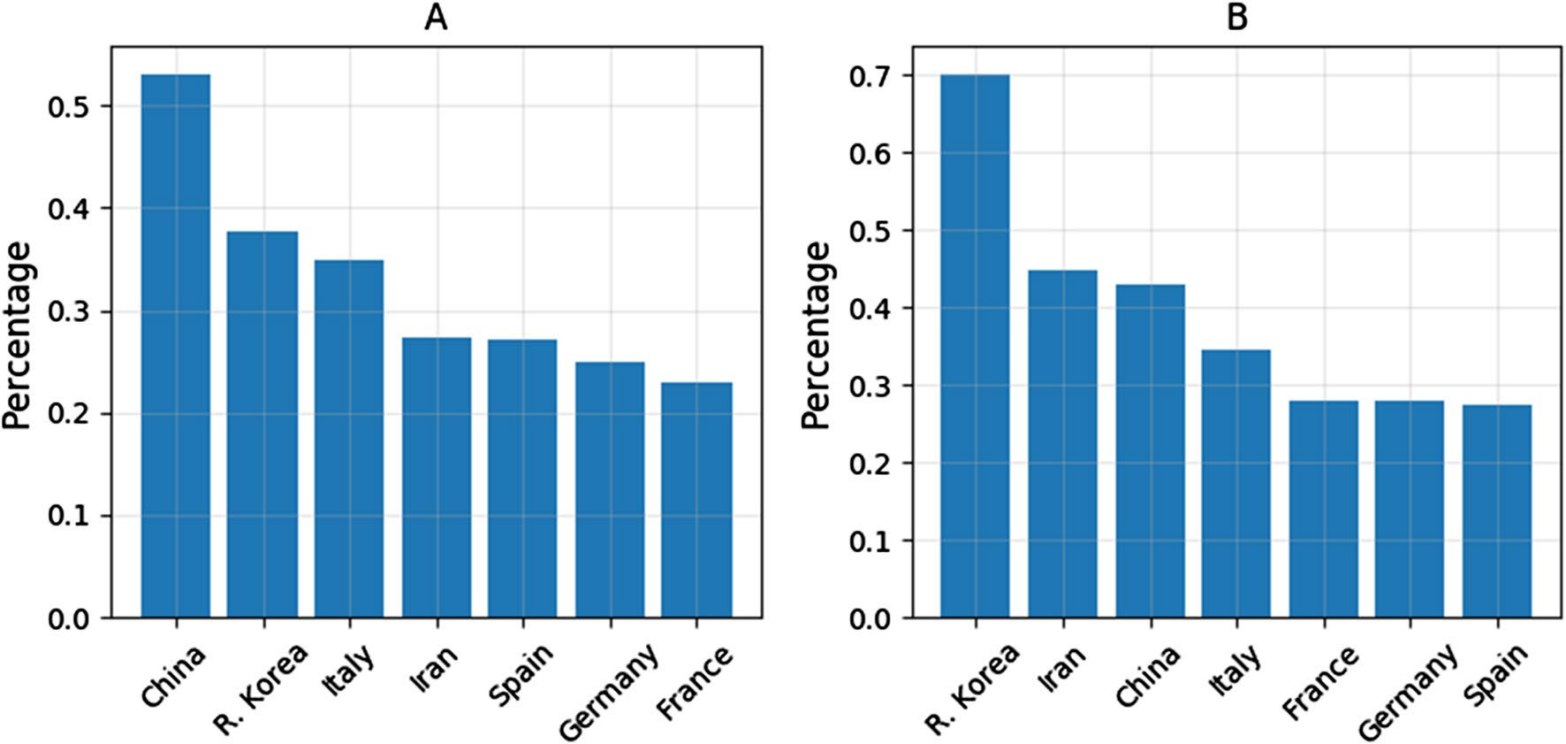
Comparisons of the total control efficiency as measured by the average proportion of decrease of the daily increase rate across different levels of weeks or cumulative cases against time (**A**) and cumulative cases (**B**) for the period of study

The total control efficiency of different countries was measured by the average proportion of decrease of daily increase rate across different level of weeks or cumulative cases (Fig. 4). For the period of the study, the rank of the total control efficiency against time was: China > R. Korea > Iran > Spain > Germany > France (Fig. 4A). The rank of the total control efficiency against cumulative cases was: R. Korea > Iran > China > Italy > France > Germany > Spain (Fig. 4B). Due to the small sample size, the t-test of control efficiency between countries is not significant except between R. Korea and France (*t* = 3.12, *p* < 0.05) against cumulative cases, and China and France (*t* = 2.49, *p* < 0.05) against time.

## Discussion

Our results indicated all seven countries showed a significant decline of daily increase rate against cumulative cases and time, indicating that the control measures, such as lockdown of the epicenter and social distancing adopted by these countries, were effective in reducing the spread of COVID-19. However, as compared to China and R. Korea, the other countries (Iran, Italy, Spain, Germany and France) showed a lower control efficiency in the later stage than before, which may have been caused by the difference in control measures at the community level (e.g. testing, tracking and isolating patients or suspected cases). Our study suggested that the daily increase rate could be useful for earlier assessment of transmission severity and trend of COVID-19, as well as the effectiveness of control measures.

Due to the highly contagious property of COVID-19, all seven countries showed a quick response to the appearance of COVID-19 cases. On January 20, soon after COVID-19 was identified as a novel coronavirus by Chinese scientists on January 7, 2020 [2, 3], the Chinese government incorporated COVID-19 into the management of statutory infectious diseases Class B and adopted prevention and control measures for Class A infectious diseases [20]. The lockdown of Wuhan (the epicenter) began on January 23 and lockdown of the whole country went into effect on January 25 [21]. Since then, travel restrictions and means for identification, isolation and observation of suspected or infected patients or places were strictly implemented. Although the effectiveness of city lockdown and travel restrictions adopted in China was questioned in the early days [4], they were later proven to be effective by the fact that China successfully contained the spread of COVID-19. Therefore, these control measures were later adopted by most countries around the world. Republic of Korea declared a state of war against the virus on March 3 [22]. Italy declared a public health emergency by January 31 and imposed nationwide lockdown on March 9 [23]. Spain declared a state of emergency on March 14 and lockdown on March 16 [24]. France declared the emergency on March 16 and lockdown on March 17 [25]. All regional governments in Germany had declared curfews or restrictions in public spaces on March 22 [26]. Iran declared city lockdowns on March 26 [27]. The significant negative associations between the daily increase rate and cumulative cases or time indicated that lockdowns and travel restrictions were effective in reducing the spread of COVID-19 in all these countries, which is consistent with the simulations in several studies [8, 11, 12]. However, as compared to China and R. Korea, Italy, Spain, Germany and France showed a lower reduction of daily increase rate after the 3rd week, which resulted in lower total control efficiency of COVID-19.

Except for city lockdowns and travel restrictions, there was a big difference in control measures taken at individual and community level in these countries. In China and R. Korea, after lockdown of cities or of the country, all patients, suspected cases and closely related people were extensively examined by testing, and isolated collectively in temporary hospitals, which helped to minimize the community transmission of COVID-19. Besides, face masks were widely used by people in China and R. Korea, which helped to minimize transmission at the individual level. In Europe, social distancing and isolation at home was widely used. Face masks were rarely used. Patients or people with close contact were not tracked extensively. Patients quarantined at home could be the significant transmission source to family members or neighbors, which likely explained the lower reduction of daily increase rate in the later stage after lockdown, and the lower total control efficiency of these countries.

As compared to the number of new cases which was widely used to judge the turning point of transmission, the daily increase rate performed better in assessing the transmission trend and control efficiency. Because number of new cases often fluctuated greatly (Fig. 1B), it was often hard to determining the turning point. Besides, it took longer (over 3 weeks) to see an obvious steadily decrease of new cases of COVID-19 for the four European countries (Fig. 1B). The steady decrease of the daily increase rate was observed within 2 weeks for all countries (Figs. 2A, 3A), thus, the daily increase rate showed a quicker response to the control measures.

The daily increase rate of cumulative cases (*r*) is a good indicator reflecting the transmission severity of a disease. From Eq. 1, for a given *r*, the double time of cumulative cases can be calculated as: T_2_ = ln (2)/*r*. For example, for *r* = 0.1, 0.2, 0.3 and 0.4, the double time of cumulative cases is 6.9, 3.4, 2.1, and 1.7 days. Therefore, COVID-19 could double its number of cumulative cases within 2–7 days if the daily increase rate is 0.1–0.4. This explains why the number of COVID-19 cases with a maximum daily increase rate around 0.25–0.39 (Table 1) could explode in a very short time. Therefore, the time-window for containing the spread of COVID-19 in its early stage is very short. Quick decisions and fast actions to take control measures is essential to contain the virus in the early stage of an epidemic.

The basic reproductive number (*R*_0_) is widely used for predicting the trend and severity of disease transmission [28]. According our previous study [16], the basic reproductive number can be estimated by the maximum daily increase rate (*r*_*m*_) and infection time of patients (IFT): *R*_*0*_ = *r*_m_*IFT. In a few recent modeling studies, the infection time of COVID-19 was often assumed to be 6 days, referring to that of SARS [10, 29], which may cause biased estimation. In our study, using published epidemiological data, the incubation time (IBT) and infection time (IFT) was estimated from the date of exposure to the virus to the date of patient hospitalization (Additional file 2: Table S1). Our estimated infection time (IFT) of COVID-19 was 8.3 ± 3.7 days (Additional file 2: Fig. S2B, Table S1), similar to the mean serial interval (i.e. the sum of the incubation period and duration of infectiveness) of a SARS-infected person (8–12 days with an average of 8.4 ± 3.8 days) in Singapore and Hong Kong [30, 31]. There was a large variation of IBT and IFT among patients of COVID-19 (Additional file 2: Fig. S2), thus, quarantine time should consider this variation. The maximum daily increase rate based on Eq. 3 for the seven countries was around 0.25–0.39 (Table 1). Thus, our estimated R_0_ of COVID-19 was from 2.1 to 3.3 using the logistic model, which was very close to that of 2.68 [10] and that of 3.11 [29].

It is notable that the early detection capacity and tests may be insufficient in some countries, resulting in low data in the early stage. Thus, we excluded data from the period of less than 100 cases from each country. However, the detection capabilities of different countries may also be different, and testing might be insufficient during epidemic periods. These problems may bring some biased estimation. Thus, it should be cautious in explaining the results of this study. Besides, our results indicate that population growth of infected patients in three countries (Italy, France and Iran) did not follow exactly the linear logistic model, instead, they followed a nonlinear logistic model (Fig. 2, Table 2). These results were caused by the poor control efficiency in the later stage in these countries. Thus, the observed convex response of daily increase rate of COVID-19 to cumulative cases suggested that more strict control measures are necessary to contain the spread of COVID-19 in these countries.

By using logistic model, we found daily increase rate was a very good indicator reflecting the transmission severity and trend of COVID-19. The maximum transmission ability of COVID-19 without human intervention can be measured by the maximum daily increase rate, while the transmission trend can be measured by the control efficiency defined in our logistic model. As compared to the other models such as SIR model or its derived models (e.g. SEIR models), the logistic model does not require knowledge of the detailed procedures of disease transmission. This is very important for assessing the transmission trend and control efficiency of COVID-19 in the early stage of disease transmission when no detailed data is often available. SIR models and its derivatives are widely used for predicting disease transmission, but they need many model parameters heavily based on detailed data of epidemiological survey. As far as we know, there is no available models to easily estimate the control efficiency of COVID-19 which would prevent us to take effective control measures in time. The change of daily increase rate as measured by the control efficiency in our logistic model is much better in reflecting the infection point of disease transmission than the widely used parameter, i.e. the number of new cases of infected patients. Therefore, our logistic model could be a complementary approach to the current disease models in assessing the trend and control efficiency of disease transmissions.

As compared to the 2002–2003 outbreak of SARS [32], COVID-19 spread much faster in both time and space. Furthermore, COVID-19 has caused a much higher number of infections and deaths than SARS in China and in the rest of the world. As compared to nearly two decades ago, the transportation capacity today is much more advanced in the world, which may partially explain why COVID-19 spread more rapidly than SARS. Revealing the transmission patterns of a disease is essential in taking effective prevention and control measures.

Under the accelerated global disease transmission we are facing in the new century, we appeal for more studies on the transmission ecology of highly contagious viruses and their influencing factors, so as to find a better solution to counter their increasing threat to public health in the modern age with advanced social and transportation networks.

## Conclusions

The logistic model is suitable to describe the transmission dynamics of COVID-19. The daily increase rate is a good indicator of reflecting the transmission severity of COVID-19. The relation of daily increase rate with cumulative cases and time could be used to assess the control efficiency. The control measures such as lockdown of epicenter, social distancing taken by different countries were effective in reducing the spread of COVID-19 but quarantine measures at a finer scale such as identification, isolation, tracking the patients and people of closely contact were essential to contain the spread of COVID-19.

## Supplementary Information

**Additional file 1: Data S1.** Data on the number of cumulative cases of COVID-19 in seven countries.

**Additional file 2: Figure S1.** An illustration on the change of number of cumulative cases (*N*_*t*_) and the number of new cases (*n*_*t*_) (A) and the relationship between daily increase rate (*r*) and number of cumulative cases of COVID-19 as described in the logistic model (modified from Zhang et al. 2004). **Figure S2.** The estimated incubation time (A) and infection time (B) of COVID-19 infected patients based on Table S1. **Table S1.** Epidemiological data of some cases of Wuhan residents and non-residents who travelled to Wuhan

## Data Availability

The datasets used and/or analysed during the current study are available from the corresponding author on reasonable request.

## Abbreviations

IBT: Incubation Time
IFT: Infection Time
R. Korea: Republic of Korea
WHO: World Health Organization
