# Supplementary material for: Modeling analysis reveals the transmission trend of COVID-19 and control efficiency of human intervention
Source: BMC Infect Dis. 2021 Aug 21;21:849. doi: 10.1186/s12879-021-06560-3 (PMC8379572; doi:10.1186/s12879-021-06560-3)
Supplement: Supplementary file 2 — Additional file 2: Figure S1. An illustration on the change of number of cumulative cases (Nt) and the number of new cases (nt) (A) and the relationship between daily increase rate (r) and number of cumulative cases of COVID-19 as described in the logistic model (modified from Zhang et al. 2004). Figure S2. The estimated incubation time (A) and infection time (B) of COVID-19 infected patients based on Table S1. Table S1. Epidemiological data of some cases of Wuhan residents and non-residents who travelled to Wuhan [file 12879_2021_6560_MOESM2_ESM.docx]

Additional Materials for

**Modeling analysis reveals the transmission trend of COVID-19 and control efficiency of human intervention**

Chaoyuan Cheng^1,3^, Xinru Wan^1^, and Zhibin Zhang^1,2^†.

Correspondence to: zhangzb@ioz.ac.cn

**This file includes:**

Figs. S1 to S2

Tables S1

Captions for Data S1


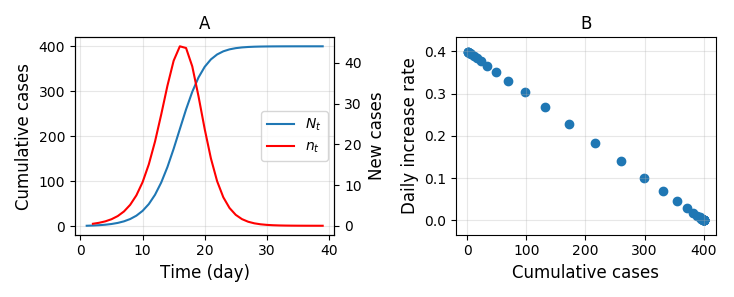


Fig. S1. An illustration on the change of number of cumulative cases (*N_t_*) and the number of new cases (*n_t_*) (A) and the relationship between daily increase rate (*r*) and number of cumulative cases of COVID-19 as described in the logistic model (modified from Zhang et al 2004). *r_t_ = a - bN_t_* (*a, b* are constants, a represents the maximum daily increase rate *r_m_*). Here, *a* = 0.4, *b* = 0.001, *N_0_* = 1.


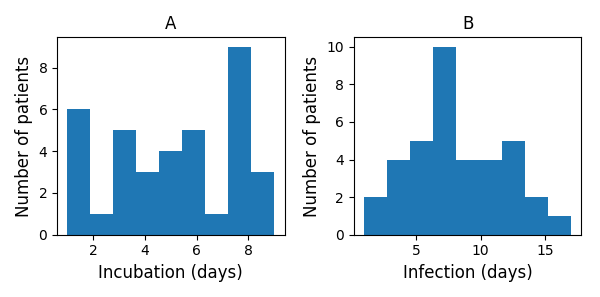


Fig. S2. The estimated incubation time (A) and infection time (B) of COVID-19 infected patients based on Table S1.

Table S1 Epidemiological data of some cases of Wuhan residents and non-residents who travelled to Wuhan. Only data of non-residents was used for estimating the incubation time (IBT) and infection time (IFT). For source of the original data, see methods section.

| Location | Date of showing symptoms | Date of being hospitalized | Arrival in Wuhan | Wuhan resident | IBT | IFT |
| --- | --- | --- | --- | --- | --- | --- |
| Shenzhen | 2020/1/4 | 2020/1/10 | 2019/12/29 | no | 6 | 12 |
| Shanghai | 2020/1/16 | 2020/1/16 | 2020/1/8 | no | 8 | 8 |
| Xicheng District | 2020/1/16 | 2020/1/20 | 2020/1/8 | no | 8 | 12 |
| Shenzhen | 2020/1/2 | 2020/1/11 | 2019/12/29 | no | 4 | 13 |
| Shenzhen | 2020/1/3 | 2020/1/10 | 2019/12/29 | no | 5 | 12 |
| Zhuhai | 2020/1/13 | 2020/1/19 | 2020/1/12 | no | 1 | 7 |
| Tongzhou District | 2020/1/19 | 2020/1/20 | 2020/1/12 | no | 7 | 8 |
| Haidian District | 2020/1/17 | 2020/1/20 | 2020/1/13 | no | 4 | 7 |
| Fengtai District | 2020/1/14 | 2020/1/20 | 2020/1/11 | no | 3 | 9 |
| Haidian District | 2020/1/9 | 2020/1/14 | 2020/1/3 | no | 6 | 11 |
| Shenzhen | 2020/1/1 | 2020/1/11 | 2019/12/29 | no | 3 | 13 |
| Pingxiang | 2020/1/10 | 2020/1/10 | 2020/1/2 | no | 8 | 8 |
| Wenzhou | 2020/1/4 | 2020/1/4 | 2020/1/3 | yes |  |  |
| Wenzhou | 2020/1/4 | 2020/1/17 | 2020/1/3 | yes |  |  |
| Fuyang | 2020/1/21 | 2020/1/21 | 2020/1/19 | no | 2 | 2 |
| Beihai | 2020/1/16 | 2020/1/17 | 2020/1/12 | yes |  |  |
| Chengdu | 2020/1/19 | 2020/1/19 | 2020/1/17 | unknown |  |  |
| Lingao County | 2020/1/16 | 2020/1/20 | 2020/1/14 | unknown |  |  |
| Xicheng District | 2020/1/20 | 2020/1/20 | 2020/1/11 | no | 9 | 9 |
| Shijingshan District | 2020/1/19 | 2020/1/21 | 2020/1/11 | no | 8 | 10 |
| Hong Kong | 2020/1/18 | 2020/1/21 | 2020/1/10 | no | 8 | 11 |
| Chaohu, Hefei | 2020/1/18 | 2020/1/20 | 2020/1/17 | yes |  |  |
| Shenyang | 2020/1/16 | 2020/1/22 | 2020/1/15 | no | 1 | 7 |
| Hefei | 2020/1/10 | 2020/1/21 | 2020/1/7 | no | 3 | 14 |
| Chizhou | 2020/1/22 | 2020/1/22 | 2020/1/19 | yes |  |  |
| Xianyang | 2020/1/20 | 2020/1/21 | 2020/1/12 | no | 8 | 9 |
| Xi'an | 2020/1/20 | 2020/1/21 | 2020/1/15 | no | 5 | 6 |
| Zhongwei | 2020/1/21 | 2020/1/21 | 2020/1/12 | no | 9 | 9 |
| Dazhou | 2020/1/18 | 2020/1/20 | 2020/1/17 | no | 1 | 3 |
| Chaoyang | 2020/1/19 | 2020/1/22 | 2020/1/18 | no | 1 | 4 |
| Lanzhou | 2020/1/18 | 2020/1/21 | 2020/1/17 | no | 1 | 4 |
| Shijingshan District | 2020/1/19 | 2020/1/21 | 2020/1/11 | no | 8 | 10 |
| Chengdu | 2020/1/18 | 2020/1/18 | 2020/1/10 | no | 8 | 8 |
| Chengdu | 2020/1/18 | 2020/1/18 | 2020/1/13 | no | 5 | 5 |
| Urumqi | 2020/1/20 | 2020/1/20 | 2020/1/15 | no | 5 | 5 |
| Shijiazhuang | 2020/1/19 | 2020/1/19 | 2020/1/18 | yes |  |  |
| Hechi | 2020/1/22 | 2020/1/23 | 2020/1/21 | yes |  |  |
| Beihai | 2020/1/22 | 2020/1/22 | 2020/1/21 | yes |  |  |
| Fuyang | 2020/1/22 | 2020/1/22 | 2020/1/19 | yes |  |  |
| Ganzhou | 2020/1/22 | 2020/1/22 | 2020/1/21 | yes |  |  |
| Hechi | 2020/1/21 | 2020/1/22 | 2020/1/15 | yes |  |  |
| Beihai | 2020/1/19 | 2020/1/19 | 2020/1/18 | yes |  |  |
| Tianjin | 2020/1/24 | 2020/1/24 | 2020/1/23 | unknown |  |  |
| Hechi | 2020/1/19 | 2020/1/21 | 2020/1/13 | no | 6 | 8 |
| Fuyang | 2020/1/22 | 2020/1/22 | 2020/1/16 | no | 6 | 6 |
| Ganzhou | 2020/1/21 | 2020/1/21 | 2020/1/17 | yes |  |  |
| Beihai | 2020/1/22 | 2020/1/22 | 2020/1/21 | yes |  |  |
| Yulin | 2020/1/21 | 2020/1/22 | 2020/1/15 | yes |  |  |
| Bozhou | 2020/1/21 | 2020/1/23 | 2020/1/19 | yes |  |  |
| Ma'anshan | 2020/1/18 | 2020/1/22 | 2020/1/16 | yes |  |  |
| Hefei | 2020/1/19 | 2020/1/22 | 2020/1/15 | no | 4 | 7 |
| Ankang | 2020/1/14 | 2020/1/23 | 2020/1/6 | no | 8 | 17 |
| Ankang | 2020/1/21 | 2020/1/23 | 2020/1/20 | yes |  |  |
| Bozhou | 2020/1/20 | 2020/1/22 | 2020/1/19 | yes |  |  |
| Hefei | 2020/1/23 | 2020/1/24 | 2020/1/22 | yes |  |  |
| Ma'anshan | 2020/1/22 | 2020/1/22 | 2020/1/20 | yes |  |  |
| Ma'anshan | 2020/1/22 | 2020/1/23 | 2020/1/18 | yes |  |  |
| Bozhou | 2020/1/19 | 2020/1/21 | 2020/1/17 | yes |  |  |
| Bozhou | 2020/1/18 | 2020/1/22 | 2020/1/16 | yes |  |  |
| Ma'anshan | 2020/1/19 | 2020/1/22 | 2020/1/16 | yes |  |  |
| Ma'anshan | 2020/1/22 | 2020/1/24 | 2020/1/16 | yes |  |  |
| Ma'anshan | 2020/1/18 | 2020/1/24 | 2020/1/9 | no | 9 | 15 |
| Huainan | 2020/1/22 | 2020/1/25 | 2020/1/19 | yes |  |  |
| Ankang | 2020/1/22 | 2020/1/22 | 2020/1/18 | yes |  |  |
| Bengbu | 2020/1/24 | 2020/1/24 | 2020/1/19 | yes |  |  |
| Macau | 2020/1/26 | 2020/1/27 | 2020/1/23 | unknown |  |  |
| Ankang | 2020/1/23 | 2020/1/23 | 2020/1/22 | no | 1 | 1 |
| Ankang | 2020/1/22 | 2020/1/24 | 2020/1/18 | yes |  |  |
| Bengbu | 2020/1/21 | 2020/1/21 | 2020/1/19 | yes |  |  |
| Hefei | 2020/1/23 | 2020/1/26 | 2020/1/22 | yes |  |  |
| Hefei | 2020/1/23 | 2020/1/26 | 2020/1/20 | yes |  |  |
| Hong Kong | 2020/1/25 | 2020/1/29 | 2020/1/22 | yes |  |  |
| Hefei | 2020/1/22 | 2020/1/23 | 2020/1/19 | no | 3 | 4 |
| Xi'an | 2020/1/21 | 2020/1/22 | 2020/1/18 | yes |  |  |
| Xi'an | 2020/1/25 | 2020/1/25 | 2020/1/20 | yes |  |  |
| Hong Kong | 2020/1/25 | 2020/1/29 | 2020/1/22 | yes |  |  |
| Hefei | 2020/1/25 | 2020/1/25 | 2020/1/20 | unknown |  |  |
| Yan'an | 2020/1/27 | 2020/1/27 | 2020/1/21 | no | 6 | 6 |
| Hefei | 2020/1/21 | 2020/1/26 | 2020/1/20 | unknown |  |  |
| Hefei | 2020/1/24 | 2020/1/27 | 2020/1/21 | unknown |  |  |
| Ankang | 2020/1/20 | 2020/1/20 | 2020/1/18 | yes |  |  |
| Ankang | 2020/1/24 | 2020/1/26 | 2020/1/19 | yes |  |  |
| Baoji | 2020/1/21 | 2020/1/22 | 2020/1/13 | yes |  |  |
| Hefei | 2020/1/30 | 2020/1/30 | 2020/1/23 | unknown |  |  |
| Hefei | 2020/1/24 | 2020/1/29 | 2020/1/21 | unknown |  |  |
| Hefei | 2020/1/30 | 2020/1/30 | 2020/1/23 | unknown |  |  |
| Hefei | 2020/1/24 | 2020/1/28 | 2020/1/22 | unknown |  |  |
| Tianshui | 2020/1/30 | 2020/1/30 | 2020/1/18 | yes |  |  |
| Xi'an | 2020/1/26 | 2020/1/28 | 2020/1/21 | yes |  |  |
| Ningxian, Qingyang | 2020/1/24 | 2020/1/28 | 2020/1/20 | yes |  |  |
| Tianshui | 2020/1/24 | 2020/1/30 | 2020/1/20 | yes |  |  |
| Tianjin | 2020/1/31 | 2020/2/2 | 2020/1/16 | unknown |  |  |
| Tianjin | 2020/1/25 | 2020/1/25 | 2020/1/5 | unknown |  |  |
| Quijing | 2020/1/26 | 2020/1/31 | 2020/1/23 | no | 3 | 8 |

**Data S1. (separate file)**

Data on the number of cumulative cases of COVID-19 in seven countries.
